# Melanotic neuroectodermal tumor of infancy: a rare presentation of an extremely rare neoplasm and diagnostic implications in Gombe, Nigeria

**DOI:** 10.11604/pamj.2017.28.5.9394

**Published:** 2017-09-04

**Authors:** Babatunde Oludare Fakuade, Joshua Biodun Adeoye

**Affiliations:** 1 Dental and Maxillofacial Department, Federal Teaching Hospital, Gombe, Nigeria; 2 Department of Preventive Dentistry, Bayero University Kano, Nigeria

**Keywords:** Melanotic neuro-ectodermal tumor of infancy, benign tumor, maxillary tumor, infant, bony-hard swelling, firm swelling, diagnostic challenges, management challenges, vanyl mandellic acid, wide excision

## Abstract

Melanotic neuro-ectodermal tumor of infancy is very rare. A unique neoplasm of the formative years, it typically involves the face or cranium; possesses rapid, expansile growth; presents as a firm swelling and displays a high rate of recurrence. Its rarity and unique features make diagnosis and management quite challenging. This challenge is increased in resource-limited settings like ours and with atypical presentation, such as was seen at our center, where patient presented with a bony hard swelling.

## Introduction

Melanotic neuroectodermal tumor of infancy (MNTI) is a rare, congenital, benign neoplasm [1-5]. It generally occurs in infancy-in the first year of life-usually as a rapidly expanding, pigmented, painless, maxillary soft tissue growth [1-5]. MNTI occasionally occurs in the skull, brain or mandible and in genital organs like epididymis, ovaries and uterus [6], however, it is most predominant in the anterior maxillary alveolus [1]. Several authors have described the tumor with different names including “Pigmented Epulis of Infancy” [2, 4], “Melanotic Progonoma” [3, 4], Melano-carcinoma and Melanotic Epithelial Odontoma, amongst others [4]. These names highlight its pigmented appearance and the existing confusion regarding its histogenesis and clinical behavior. The name “MNTI” is now most accepted as it accurately reflects the unique aspects of its presentation-pigmentation caused by melanin [1-3]; occurrence during infancy; and development from neural crest cells [1, 5]. In a histological review, Dehner et al [5] described various stages of melanocyte and neuroblast-like cells found in MNTI cases and suggested a neuro-ectodermal origin [2-4]. They are regarded as benign tumors and are often associated with displacement of developing or erupted teeth [1, 2, 7]. They can however be locally aggressive, invading surrounding bones and sinuses and this may explain their high recurrence rate, reported to be between 15-20% [1, 7, 8] or as high as 50% in cases without wide resection [3]. They also usually present as firm swellings, as they have a soft tissue origin. While clinical and radiological findings may suggest a diagnosis of MNTI, histo-pathologic examination is required for definitive diagnosis [3]. This is because it has features in common with other tumors such as retinoblastoma and phaeochromocytoma [1]. In addition to its diagnostic challenges, MNTI also poses a management challenge to clinicians due to its high recurrence rate, often-dramatic presentation and occurrence during infancy. All of these are worsened in resource-limited settings where one or more of the facilities for diagnosis or management may be absent. There is no report of this tumor from the North Eastern region of Nigeria. We present a case of MNTI still being followed 2 years after surgery.

## Patient and observation

**Figure 1:**
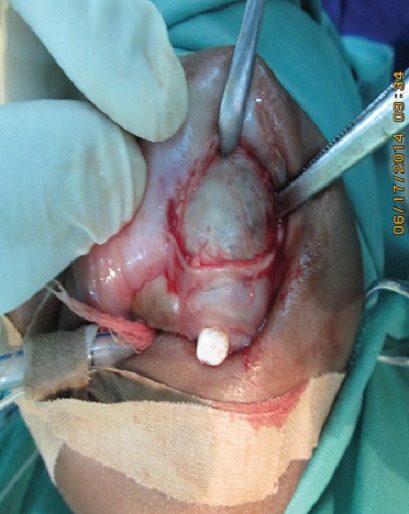
Clinical intraoral photograph showing exposed tumor in the maxillary region

**Figure 2:**
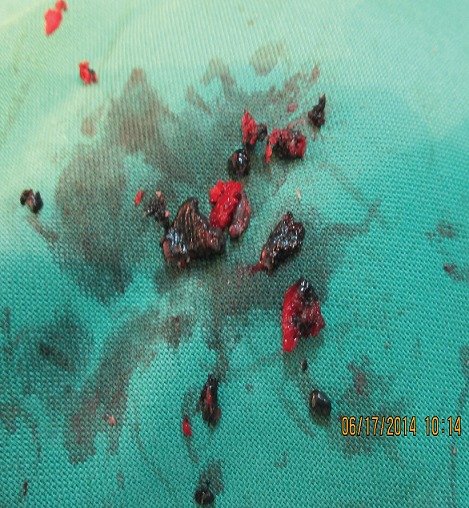
Pigmented granules seen during surgery

**Figure 3:**
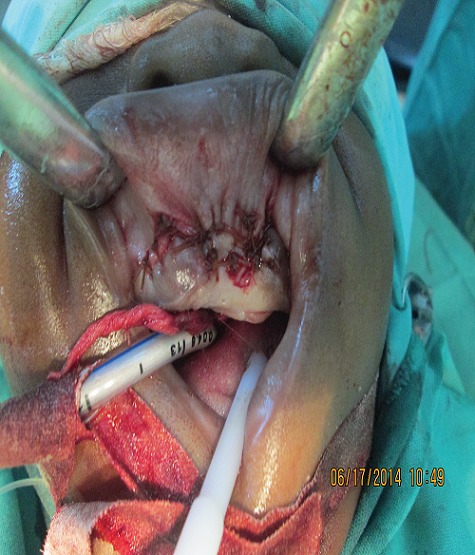
Post-operative photograph after wide excision

**Figure 4:**
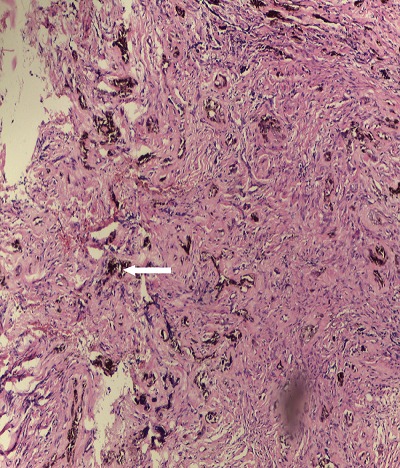
Histological section of the lesion showing nest of pigmented cells (arrow) within the connective tissue (H&E x10)

**Figure 5:**
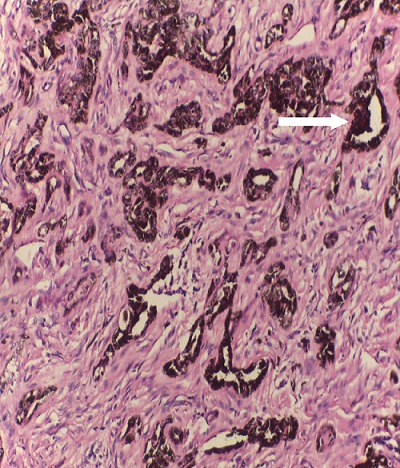
Histological section showing nest and island of tumor cells with cleft formation (arrow) (H&E x40)

**Figure 6:**
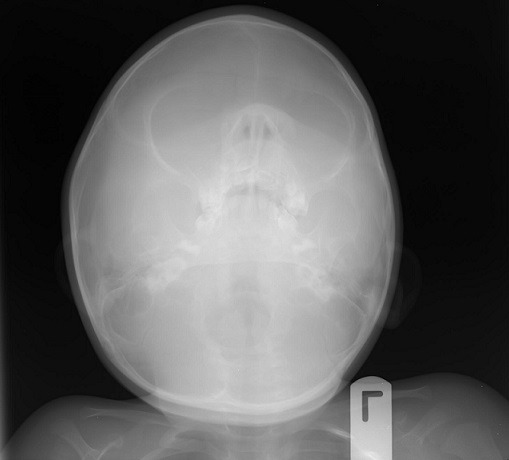
Occipito-mental view of the skull showing anterior-maxillary region

**Figure 7:**
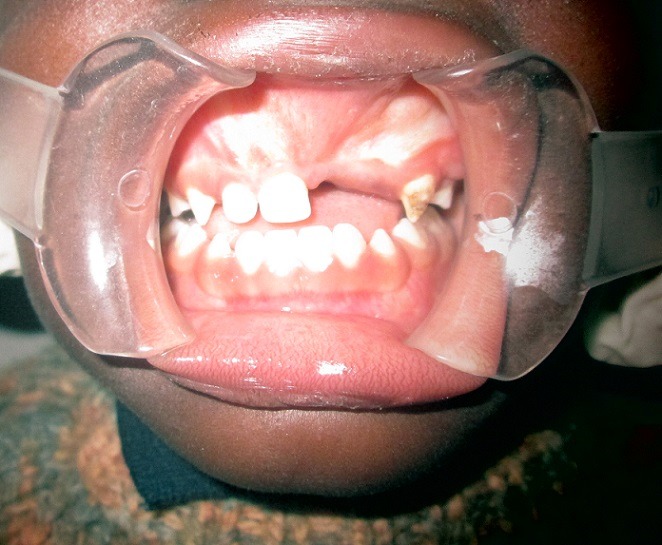
Patient 2 years post-excision

A 6-month old male child was referred to the unit on account of an expansile maxillary growth that started from birth and had progressively increased in size. The swelling was painless and did not obstruct breathing. The mother however, sought care due to compromised feeding and cosmesis. She had attended antenatal care in a village cottage hospital nearby and volunteered normal birth, family and immunization history. There was also no history of medication use during pregnancy. She had been referred from this center to ours after the birth of the baby. Examination revealed marked facial asymmetry caused by a non-tender, non-pulsatile, bony-hard mass in an otherwise healthy child. The swelling crossed the midline, extending from the right canine region to the left incisor region of the gum-pad (Figure 1), measuring 3cm by 4cm. The overlying mucosa had a bluish tinge. 150 and 300 occipito-mental views on plain radiographs revealed a radiolucency of the left maxillary region, with the maxillary antra clear. A working diagnosis of “congenital osteoma” replaced an initial impression of “dentigerous cyst” and the child was scheduled for excisional biopsy under general anesthesia due to a lack of cooperation with attempts at an FNAC or an incisional biopsy. Due to the child's compromised feeding before presentation, with blood work revealing anisocystic anemia and a Packed Cell Volume (PCV) of 24%, he was placed on double-dose hematinics, high-protein diet and broad-spectrum antibiotics for two weeks. By the end of the build-up period, the PCV was about 30% and he was scheduled for surgery. Intra-operatively, access was gained through the labial mucosa, revealing blackish granules (Figure 2). The operator decided against a Weber-Ferguson approach as cosmesis was important to the mother and the labial approach provided good access (Figure 3). The exposed tumor contained an erupted primary central incisor in close proximity to an un-erupted tooth (possibly the permanent successor). The erupted tooth was removed while the un-erupted tooth was left in situ. To ensure total excision, the excisional biopsy was followed by a peripheral ostectomy of a 5mm margin. As primary closure could be achieved and as the nasal orifices were patent, the surgeons decided against taking a palatal impression for a feeding plate or nasal stents. Histopathology of the excised tissue revealed pigmented cells in nests and islands, with some cleft formation (Figure 4, Figure 5). Taken with the intraoperative findings, a diagnosis of MNTI was reached. Due to the high recurrence rate of the tumor, the child was followed up every three months for the first year, then annually afterwards, in line with regimens worldwide. There is currently no evidence of recurrence at two years following resection (Figure 6, Figure 7).

## Discussion

Melanotic neuroectodermal tumor of infancy is a rare, benign, congenital neoplasm commonly seen in infants with a variable age of occurrence [1-9] and a lot of reports report presentation of the infants at onset. For example, Borello and Gorlin [4] reported a case with onset of 3 months while Mummery and Pit [10] reported a case at 5 months. It is instructive that our patient was seen at 6 months of age despite occurrence of the tumor at birth. Ajagbe et al [11] and Williams [12] in case reports on MNTIs from Ibadan, Nigeria noticed a similar trend. The 2 cases reported by Ajagbe presented at 6 months and the case reported by Williams presented at 8 months. It is an indicator of poor attitudes towards healthcare in Nigeria and most developing countries [13]. By her admission, the mother was only motivated to seek care when feeding was affected and cosmetic compromise was marked. The trend is also baffling because the child was birthed in a hospital, albeit a village cottage one. One would expect that with onset at birth, and with the prominent site of occurrence-the anterior maxilla, similar to most literature that report that 70% of cases occur here [1, 14], the wait would be shorter. Like most reported cases of MNTI, our patient presented with a painless swelling. MNTI is generally painless [1-4] unless there is secondary infection or with advanced tumors. Since the painlessness seemed to contribute to the late presentation, as part of management and follow-up, the mother was counseled on seeking care appropriately and not just with pain or marked deformity. Establishing the diagnosis of MNTI in this case highlights the diagnostic challenges inherent, especially with limited radiographic investigative tools available in settings like ours. While greater than 90% of MNTI cases present during the first year of life [1-4], so do 90% of congenital epulis, for example [1, 15]. MNTIs have no gender predilection [3, 6] or slightly favor males with 1.48 to 1 [7]; but so do congenital eruption cysts [16]. The clinical presentation of a bony-hard swelling as seen in this case is rarely reported [1-7]; and is instead expected for tumors such as osteomas or fibrous dysplasia. These tumors therefore remained valid differentials and helped inform the decision to investigate using plain radiographs.

Radiology often reveals a destructive, poorly demarcated radiolucency of the underlying bone with a “sunburst” appearance from mild calcification along vessels radiating from the center of the tumor [9]. This was also not seen in this case (Figure 7), abetting the confusion in diagnosis. An MRI or CT scan may have been of more diagnostic use, as MNTI is a soft tissue tumor. These diagnostic modalities were not available, however. Even though they are regarded as benign, MNTI may cause destruction of bone and displacement of teeth [1]. This is because they can be locally aggressive, invading surrounding bones and sinuses [1]. In this case however, the bony borders of the maxillary antra were spared and most of the tumor growth was extra-oral. The tumor also did not displace the spared, un-erupted tooth. This probably contributed to the non-specific findings on the plain radiographs taken. While an FNAC or an incisional biopsy would have revealed high levels of Vanyl Mandelic acid (VMA), seen in MNTIs, it is important to note that VMA is also found in retinoblastoma, ganglio-neuroblastoma, or pheochromocytoma [2-4] and is therefore not pathognomonic of MNTIs. Borello and Gorlin [4] in 1966 were the first to report on the high urinary excretion of VMA, suggesting its neural crest origin and proposing the name MNTI. The bony consistency of the tumor in this patient and his uncooperativeness complicated access to the tumor however. The lesion is usually solitary with intact mucosa, typically made bluish by the presence of melanin in the lesion [5]. The present case had the typical bluish presentation, in addition to the pigmented granules seen intra-operatively. Ultimately, the bluish-blackish granules revealed by the labial incision in the excisional biopsy confirmed MNTI as the working diagnosis. Prescribed treatment includes radical surgery [3, 6,14] and wide surgical excision with ostectomy [1] for new lesions. Radical surgery with adjuvant treatment is suggested for recurrent lesions [3, 17]. All of these have been prescribed because of its high recurrence rate, which is reported in some cases to occur just few weeks post-operation [8]. Carnevale et al [18] even suggested the use of operating microscopes during surgical excision to remove unseen remnants of the pigmented lesion to prevent its recurrence. Recurrence is also reported to be commoner amongst younger patients [14, 17] although this may be because it is easier to be more radical when there is more tissue to manipulate, as would naturally occur in older patients. We decided on less radical surgery in this case and performed a peripheral ostectomy of 5mm with the surgical excision. Follow up of this case 24 months later shows no recurrence. The spared tooth, and the lack of an extra-oral incision scar have also yielded a pleasing result.

## Conclusion

We have presented a rare tumor in a 6-month-old infant in a tertiary hospital in North Eastern region of Nigeria-a melanotic neuroectodermal tumor of infancy, with a rare presentation-a bony-hard swelling as opposed to the normal presentation of a firm swelling. The delayed presentation by the mother until feeding was compromised also made the case noteworthy. The diagnostic and management challenges in such a situation, related to resource challenges, is also presented.

## Competing interests

The authors declare no competing interests.
